# Bromelain and N-acetylcysteine (BromAc) as novel adjunctive therapy for prosthetic joint infection: a narrative review

**DOI:** 10.5194/jbji-11-441-2026

**Published:** 2026-07-28

**Authors:** Brendan Parnell, David Morris

**Affiliations:** 1 Department of Orthopaedic Surgery, St George Hospital, Kogarah NSW 2217, Australia; 2 Peritonectomy Unit, Department of Surgery, St George Hospital, Kogarah NSW, 2217, Australia; 3 St George Hospital Clinical School, University of New South Wales, Kogarah NSW, 2217, Australia

## Abstract

**Background**: Prosthetic joint infection (PJI) complicates 0.5 %–2.0 % of total joint arthroplasties and remains the leading cause of implant failure. Bacterial biofilm confers marked antimicrobial resistance, and current strategies, including debridement, antibiotics, and implant retention (DAIR), carry a pooled failure rate of ∼ 36 %. Novel biofilm-disrupting adjuncts are urgently needed. **Purpose**: The aim is to examine preclinical evidence of bromelain and N-acetylcysteine (BromAc) as a combination antibiofilm therapy in PJI management and to critically appraise its translational readiness. **Methods**: A narrative literature search was conducted for studies examining N-acetylcysteine, bromelain, and BromAc in the context of biofilm disruption on orthopaedic prosthetic materials. Eligible studies were appraised qualitatively for pathogen, substrate, exposure conditions, outcome measures, and methodological limitations to allow comparative interpretation rather than narrative summary alone. **Results**: N-acetylcysteine demonstrates concentration-dependent antibiofilm activity, achieving approximately 50 % biofilm reduction on polyethylene and 20 % on titanium at minimum inhibitory concentrations, rising to 81.5 % eradication of staphylococcal biofilms at higher concentrations in non-orthopaedic substrates. Bromelain achieves significant biofilm reduction from orthopaedic hardware via proteolytic degradation of extracellular polymeric substance protein scaffolds. In the principal proof-of-concept study on hernia mesh, BromAc achieved > 80 % biofilm removal (quantified by crystal violet biomass reduction) across three *Pseudomonas aeruginosa* strains, exceeding either agent alone; activity against staphylococci has been reported in unpublished sponsor data but is not yet independently replicated in peer-reviewed orthopaedic models. **Conclusions**: BromAc represents a mechanistically rational adjunctive therapy for PJI, targeting distinct biofilm matrix components. However, current evidence is largely preclinical, much of it derived from non-orthopaedic substrates and pathogens, and several supporting datasets are unpublished sponsor data. Important translational gaps remain in peer-reviewed staphylococcal validation on prosthetic materials, formulation stability (particularly the susceptibility of N-acetylcysteine to oxidation in aqueous solution), intra-articular safety with respect to cartilage and osseointegration, and the absence of an in vivo orthopaedic implant model. These limitations must be resolved before clinical translation can be responsibly considered.

## 1 Introduction

Prosthetic joint infection (PJI) represents one of the most serious complications following total joint arthroplasty, with a reported incidence ranging from approximately 0.5 %–2 % following primary joint arthroplasty (Aftab et al., 2025; Perni and Prokopovich, 2024; Weinstein et al., 2023). With over 1 million total joint arthroplasties performed annually worldwide, PJI affects tens of thousands of patients yearly, creating a substantial public health burden (Aftab et al., 2025; Tande and Patel, 2014).

PJI is defined by pathogenic microorganisms within the joint space or on the prosthetic implant surface, leading to inflammation, tissue destruction, and potential systemic infection (Tande and Patel, 2014). The Infectious Diseases Society of America (IDSA) establishes diagnostic criteria and management guidelines that form the foundation of current practice (Osmon et al., 2013). Despite advances in surgical technique and antimicrobial prophylaxis, PJI remains the leading cause of failure following total hip and knee arthroplasty (Patel, 2023; Tande and Patel, 2014).

Temporal classification divides infections into early ( < 3 months), delayed (3–12 months), or late ( > 12 months) based on microbiological profiles (Tande and Patel, 2014). Early infections typically involve *Staphylococcus aureus*, while delayed and late infections may involve coagulase-negative staphylococci or *Cutibacterium acnes* (Tande and Patel, 2014; Weinstein et al., 2023).

The healthcare burden is substantial. Infected patients require prolonged hospitalisation, multiple surgical procedures, and extended antimicrobial therapy and frequently experience permanent functional impairment (Aftab et al., 2025; Patel, 2023). Treatment failures necessitate prosthesis removal with options including two-stage revision, debridement with implant retention (DAIR), or resection arthroplasty (Abbaszadeh et al., 2026). Even with aggressive management, functional outcomes are often inferior to uncomplicated primary arthroplasty (Gehrke et al., 2024).

N-acetylcysteine (NAC), bromelain, and their fixed combination (BromAc^®^, Mucpharm Pty Ltd) are introduced here at the outset so that subsequent sections on biofilm pathophysiology and current management can be read in light of the proposed adjunct. NAC is the N-acetylated derivative of L-cysteine, and has been established in clinical use for decades as a mucolytic, as the antidote for paracetamol poisoning, and as a renal-protective agent; its free thiol group reduces disulfide bonds within mucopolysaccharide and biofilm matrices, conferring antibiofilm activity in vitro at concentrations achievable by local rather than systemic delivery (Blasi et al., 2016; Oliva et al., 2023).

Bromelain is a mixture of cysteine proteases extracted from pineapple (*Ananas comosus*) stem, used clinically as an anti-inflammatory and wound-debridement agent, with regulatory precedent in the form of NexoBrid^®^ for enzymatic burn debridement. Its proteolytic activity targets the protein scaffold (including fibrin and collagen) that stabilises biofilm architecture (Chisci and Fredianelli, 2022; Kansakar et al., 2024; Rathnavelu et al., 2016; Watters et al., 2016).

BromAc is a co-formulated combination of bromelain with NAC, developed by Mucpharm Pty Ltd as a mucolytic-/biofilm-disrupting agent. It has progressed through a first-in-human and Phase III trial in pseudomyxoma peritonei, where it is delivered by intratumoural or intraperitoneal injection (Mucpharm, data on file). To date, no published human data exist for orthopaedic or intra-articular use, and BromAc is not currently approved for PJI in any jurisdiction. The rationale for examining it in this context is mechanistic: PJI biofilms contain both polysaccharide-/disulfide-stabilised and proteinaceous components and a dual-action agent that simultaneously targets both; is already supported by safety data in other intracavitary applications; and is an a priori attractive adjunct to DAIR, where biofilm persistence is the dominant driver of failure.

This review therefore (i) summarises the pathophysiology of PJI biofilm and the limits of current management; (ii) appraises the preclinical evidence base for NAC, bromelain, and BromAc with explicit attention to substrate, pathogen, exposure, outcome metric, and study quality; and (iii) defines the translational gaps, staphylococcal validation on prosthetic materials, formulation stability, intra-articular safety, and orthopaedic in vivo evidence that must be closed before any clinical evaluation in PJI.

## 2 Methods

A narrative literature search was conducted in January 2026 using PubMed/MEDLINE to identify relevant studies examining prosthetic joint infection (PJI), N-acetylcysteine (NAC), bromelain, and combination biofilm-targeting therapies. Four separate structured searches were performed using Medical Subject Headings (MeSH) terms and Boolean operators: (1) (“prosthetic joint infection”[tiab] OR “periprosthetic joint infection”[tiab]) AND (epidemiology[tiab] OR burden[tiab] OR incidence[tiab] OR pathogenesis[tiab] OR biofilm[tiab] OR review[pt]), filtered for review articles to capture PJI background literature; (2) (acetylcysteine[mesh] OR “N-acetylcysteine”[tiab] OR “N-acetyl-L-cysteine”[tiab]) AND (biofilm[tiab] OR “prosthetic joint”[tiab] OR periprosthetic[tiab] OR orthopedic[tiab] OR orthopaedic[tiab]) for NAC and orthopaedic infection studies; (3) (“bromelain”[tiab] OR “BromAc”[tiab] OR “BromNac”[tiab]) AND (biofilm[tiab] OR “prosthetic”[tiab] OR implant[tiab] OR orthopedic[tiab] OR orthopaedic[tiab]) for bromelain and implant biofilm studies; and (4) (“bromelain”[tiab] AND (“N-acetylcysteine”[tiab] OR “acetylcysteine”[mesh])) AND biofilm[tiab] for combination therapy studies. Studies were included if they were peer-reviewed English-language publications examining PJI epidemiology and management, NAC or bromelain activity against biofilms on prosthetic materials, or combination therapy for biofilm-related infections. Priority was given to systematic reviews, clinical guidelines, and studies using clinically relevant PJI pathogens (*S. aureus*, coagulase-negative staphylococci, *P. aeruginosa*) tested on actual prosthetic materials (titanium, polyethylene).

Where unpublished sponsor data (Mucpharm Pty Ltd, “data on file”) are cited, this is identified explicitly in the text. These reports were not peer reviewed, and their inclusion is restricted to indicating the direction of currently available evidence; conclusions drawn from them are accordingly qualified. The competing interest associated with these sources is declared in the “Competing interests” statement.

## 3 Biofilm pathophysiology and current management limitations

The central pathophysiological feature of PJI is bacterial biofilm formation on the prosthetic implant surface (Patel, 2023; Tande and Patel, 2014). Biofilms are structured microbial communities encased within an extracellular polymeric substance (EPS) matrix composed of polysaccharides, extracellular DNA, proteins, and lipids (Tande and Patel, 2014). This matrix creates a protective microenvironment conferring marked resistance to antimicrobial agents and host immune defences (Patel, 2023; Tande and Patel, 2014).

Bacteria within biofilms demonstrate reduced susceptibility to antibiotics compared to planktonic counterparts (Patel, 2023; Tande and Patel, 2014). Multiple mechanisms contribute to biofilm-mediated resistance, including (1) impaired antimicrobial penetration by the EPS barrier, (2) metabolic heterogeneity with slow-growing dormant bacteria, (3) persister cell formation displaying extreme antibiotic tolerance, (4) altered microenvironment with pH gradients and oxygen depletion, and (5) horizontal gene transfer promoting resistance (Patel, 2023; Tande and Patel, 2014). Common PJI pathogens, including *Staphylococcus aureus*, coagulase-negative staphylococci, and *Pseudomonas aeruginosa*, are particularly adept at biofilm formation (Patel, 2023; Tande and Patel, 2014).

Current treatment paradigms emphasise early intervention, aggressive surgical debridement, and prolonged antimicrobial therapy (Osmon et al., 2013). DAIR procedures aim to preserve the prosthesis through thorough debridement, component exchange when feasible, and targeted antibiotics (Longo et al., 2024). In this review, the term “debridement”, as applied to BromAc and other in vitro studies, refers to the percentage reduction in adherent biofilm biomass (typically measured by crystal violet optical density or colony-forming unit recovery from sonicated implants) rather than to clinical/operative tissue resection. Where authors use the term “eradication”, we have retained the original authors' definition (most commonly the absence of recoverable colony-forming units after exposure) and have flagged where eradication is inferred from biomass-only assays. Studies have shown DAIR failure rates to vary widely from 0 %–84.4 %, with a pooled estimate of 35.9 % (Abbaszadeh et al., 2026). This depends on patient factors; organism virulence; and, critically, the timing of intervention, with acute infections showing significantly lower failure rates (34.2 %) compared to late chronic infections (73.6 %) (Abbaszadeh et al., 2026). The fundamental problem is that conventional surgical debridement cannot reliably eliminate biofilm from prosthetic surfaces (Izakovicova et al., 2019). Mechanical debridement with curettes and pulse lavage achieves incomplete biofilm disruption, and systemic antibiotics penetrate biofilms poorly (Izakovicova et al., 2019).

Two-stage revision with prosthesis removal, antibiotic spacer placement, and delayed reimplantation has been shown to achieve higher success rates, typically ranging from 54 %–100 %, with some studies reporting up to 100 %, though results vary considerably based on patient selection and definition of success (Bourgonjen et al., 2021).

However, two-stage revision requires multiple surgeries, prolonged disability, substantial cost, and permanent soft tissue compromise (Gehrke et al., 2024; Kini et al., 2016; Qin et al., 2024). This creates an urgent need for adjunctive therapies capable of disrupting biofilm architecture and enhancing antimicrobial penetration to improve DAIR success rates and avoid prosthesis removal.

## 4 Emerging antibiofilm strategies for implant-associated infections

The limitations of conventional antibiotic-based therapy have driven investigation into diverse antibiofilm strategies, broadly classified into enzymatic degradation of the EPS matrix, chemical and small-molecule disruption, physical and electrochemical methods, and surface modification (Scalia and Najmi, 2025; Visperas et al., 2022).

Biofilm-dispersing enzymes directly degrade structural components of the EPS matrix and represent one of the most promising approaches to biofilm eradication (Al-Madboly et al., 2024; Wang et al., 2023). Three principal enzyme classes have been investigated:

*Glycoside hydrolases*, most notably dispersin B (DspB), which degrade poly- β (1,6)-N-acetylglucosamine (PNAG), detach preformed biofilms, and sensitise bacteria to antibiotics and antiseptics across both Gram-positive and Gram-negative pathogens. A wound gel formulation (DispersinB^®^, Kane Biotech) has progressed to human clinical trials (Kaplan et al., 2024; Wang et al., 2023). *DNases* target extracellular DNA, reducing biofilm biomass and antibiotic tolerance, with recombinant DNase (Dornase alfa) already approved for cystic fibrosis, providing a regulatory precedent. Efficacy is greatest against immature biofilms (up to 60 h) (Wang et al., 2023). *Proteases*, including combinations of endolysins (M23, GH15) with the depolymerase DA7, significantly improve antibiotic efficacy against *S. aureus* and *S. epidermidis* biofilms (Wang et al., 2023). A critical principle is that enzymatic dispersal must be co-administered with antimicrobials, as dispersal alone may release viable bacteria and worsen disseminated infection (Wang et al., 2023).

Chemical and small-molecule agents include quorum sensing (QS) inhibitors (e.g., 5-fluorouracil), which disrupt biofilm maturation signalling but are limited by toxicity; antimicrobial peptides (AMPs) such as LL-37 and oritavancin, which provide broad-spectrum antibiofilm activity through membrane disruption; nitric oxide (NO)-releasing compounds and nitroxide-functionalised antibiotics (e.g., CTEMPO-ciprofloxacin), which combine biofilm dispersal with bactericidal activity; and cyclic-di-GMP/c-di-AMP modulators under preclinical investigation (Visperas et al., 2022).

Physical and electrochemical approaches include cathodic voltage-controlled electrical stimulation (CVCES), which generates reactive oxygen species and pH shifts to reduce viable biofilm bacteria; controlled hyperthermia, which releases biofilm-embedded organisms and enhances antibiotic susceptibility; and antimicrobial photodynamic therapy (aPDT), which uses light-activated photosensitisers to generate cytotoxic ROS within the biofilm (Scalia and Najmi, 2025; Visperas et al., 2022).

Surface modification strategies, including antimicrobial coatings (silver, copper, zinc oxide nanoparticles), hydrogel surfaces, and titanium nanotopographic modifications, are primarily preventive rather than therapeutic, reducing initial bacterial adhesion and thereby the biofilm burden requiring subsequent eradication (Scalia and Najmi, 2025).

## 5 N-acetylcysteine: mechanisms and orthopaedic biofilm evidence

N-acetylcysteine (NAC) is the acetylated derivative of L-cysteine and a precursor to glutathione biosynthesis. Clinically used for decades as a mucolytic agent in respiratory conditions and acetaminophen antidote, NAC demonstrates significant antibiofilm activity through complementary mechanisms (Blasi et al., 2016; Oliva et al., 2023).

The primary antibiofilm mechanism involves thiol-mediated disruption of disulfide bonds within the EPS matrix (Blasi et al., 2016). NAC's free thiol group (-SH) reduces disulfide crosslinks that stabilise biofilm polysaccharides and proteins, thereby destabilising matrix architecture and facilitating disaggregation (Blasi et al., 2016). Secondary mechanisms include inhibition of bacterial adhesion to respiratory epithelial cells and surfaces, perturbation of intracellular redox equilibrium and bacterial metabolism (Blasi et al., 2016), and direct antimicrobial activity against planktonic bacteria at concentrations ≥ 5–40 mg mL^−1^ (Blasi et al., 2016).

The landmark study by Drago et al. (2013) directly examined NAC activity against biofilms of *S. aureus* and *P. aeruginosa* grown on orthopaedic prosthetic materials (Drago et al., 2013). Using smooth polyethylene and sand-blasted titanium discs, the materials employed in actual hip and knee prostheses, investigators demonstrated that NAC achieves concentration-dependent biofilm reduction on prosthetic materials, with approximately 50 % biofilm eradication on polyethylene surfaces, with lower efficacy (approx. 20 %) on titanium at MIC-range concentrations after 3 h of exposure. Reduction in this study was quantified by crystal violet biomass; the assay does not distinguish viable from non-viable bacteria, and the 3 h exposure is shorter than typical operative DAIR contact times, both of which limit direct translation to the operating theatre. More recent studies in non-orthopaedic settings have expanded upon this work, documenting that NAC at 24.8 mg mL^−1^ ( 4× MIC) achieved complete eradication in 42.6 % of isolates, increasing to 81.5 % of isolates at 49.6 mg mL^−1^ ( 8× MIC) in *Staphylococcus* isolates from chronic rhinosinusitis (Jotic et al., 2025). These rhinosinusitis isolates are clinically distinct from PJI isolates in adhesion phenotype, substrate (mucosal rather than metallic/polymeric), and exposure environment; the figures should therefore be interpreted as supportive rather than directly transferable. Combination treatments incorporating 30 mM NAC with antibiotics achieved ≥ 90 % biofilm disruption across MRSA and MSSA strains (Manoharan et al., 2020). These concentrations are potentially clinically achievable through direct intra-articular administration, given that precedent from intra-articular antibiotic protocols demonstrates achievement of local synovial concentrations much higher than systemic bioavailability (Steadman et al., 2023). In contrast, systemic bioavailability following oral NAC administration remains limited (4 %–11.6 % due to extensive first-pass hepatic metabolism), underscoring the rationale for local rather than systemic delivery to achieve therapeutic biofilm-disrupting concentrations (Teder et al., 2021; Tenório et al., 2021).

Other translational research has explored NAC incorporation into antibiotic-loaded bone cement (Tseng et al., 2025). These formulations demonstrated enhanced bactericidal activity against common PJI pathogens (*S. aureus*, *E. coli*) in both planktonic and biofilm states. While NAC incorporation can reduce cement mechanical properties, formulations combining NAC with teicoplanin-loaded cement maintain mechanical resistance within acceptable limits, suggesting potential for clinical application with optimised antibiotic combinations (Tseng et al., 2025).

## 6 Bromelain: enzymatic debridement of infected implants

Bromelain is a complex mixture of proteolytic enzymes (primarily cysteine proteases) derived from pineapple stem. Used clinically as an anti-inflammatory agent and wound debridement aid, bromelain's broad proteolytic activity breaks down proteins and peptide bonds, degrading proteinaceous components of biofilm matrices (Chisci and Fredianelli, 2022; Kansakar et al., 2024; Rathnavelu et al., 2016).

Bromelain's cysteine protease activity specifically targets peptide bonds in the protein scaffold of the EPS matrix, with documented efficacy against both fibrin and collagen components that stabilise biofilm architecture (Watters et al., 2016). In a wound-relevant *S. aureus* biofilm model supplemented with human plasma, bromelain reduced biofilm biomass by up to 98 % (Watters et al., 2016). It should be emphasised that this 98 % figure was obtained on plasma-supplemented in vitro biofilm in a wound-care model and is not equivalent to the multilayered, shear-protected biofilm that forms on a loaded titanium or polyethylene prosthesis in vivo.

Bratton et al. (2025) assessed the efficacy of bromelain in debriding MRSA biofilm from cortical bone screws using crystal violet staining analysed by optical density (OD), with six screws per treatment group. Low-dose bromelain solution alone did not significantly reduce biofilm compared with controls (OD 0.104 ± 0.047; p=0.345 ). However, low-dose bromelain with scrubbing (OD 0.068 ± 0.020; p=0.012 ) and high-dose bromelain with scrubbing (OD 0.045 ± 0.014; p=0.001 ) both demonstrated significant biofilm reduction. High-dose bromelain solution without scrubbing (OD 0.056 ± 0.012; p=0.003 ), bromelain powder alone (OD 0.041 ± 0.010; p=0.001 ), and bromelain powder with scrubbing (OD 0.032 ± 0.005; p<0.0001 ) all yielded significantly lower OD values than their respective controls, with the powder-plus-scrubbing group achieving the greatest biofilm reduction. These findings suggest bromelain is a promising enzymatic debridement agent for biofilm-contaminated orthopaedic implants (Bratton et al., 2025). An important methodological limitation of Bratton et al. (2025) is that crystal violet optical density measures total adherent biomass (live and dead cells together with residual matrix) and does not distinguish viable from non-viable organisms; reduction in OD therefore evidences biofilm disaggregation rather than bactericidal activity. The study also used cortical bone screws rather than a loaded titanium or polyethylene prosthesis, employed a single MRSA strain, and used short in vitro exposure times. Reduction in OD must therefore not be over-interpreted as bacterial eradication, and corroborating CFU recovery and live/dead viability assays will be required in any further work.

## 7 Pharmacokinetic and formulation stability considerations

Any clinical translation of NAC, bromelain, or BromAc to intra-articular or perioperative use is conditional on the demonstrated stability and pharmacokinetic behaviour of the active agents at the site of action. NAC is well recognised to be unstable in aqueous solution: the free thiol group that confers antibiofilm activity is also susceptible to autoxidation to N,N^′^-diacetylcystine, with the rate of oxidation increasing in the presence of oxygen, metal ions, elevated pH, and elevated temperature. Reported shelf lives of reconstituted NAC solutions range from a few hours under ambient conditions to up to 24 h under refrigeration and protection from light, depending on buffer composition and antioxidant additives. Loss of free thiol translates directly into loss of biofilm-disrupting activity. In an operative DAIR context, this implies that BromAc cannot be assumed to retain full activity if reconstituted in advance or stored open in theatre and that point-of-use mixing protocols, preservative-free formulation, and oxygen-protective packaging will likely be required. Bromelain in aqueous solution is also subject to time- and temperature-dependent loss of proteolytic activity through autodigestion; commercial preparations are typically stabilised and lyophilised. Synovial fluid pharmacokinetics of either agent after intra-articular delivery are unknown, including residence time, clearance, and protein binding within an infected joint; these data will be required before dose, contact time, and re-dosing intervals can be rationally specified. Together, these formulation considerations represent a translational barrier that is independent of, and in addition to, the question of biological efficacy.

## 8 BromAc combination therapy: rationale and preclinical evidence

The combination of bromelain and N-acetylcysteine (BromAc) represents a mechanistically rational approach by targeting distinct EPS components (Carter et al., 2021). NAC targets a polysaccharide matrix through thiol-mediated disruption (Blasi et al., 2016), while bromelain is a proteolytic enzyme (Varilla et al., 2021) which has been shown to have effectiveness in enzymatic degradation of biofilms (Watters et al., 2016). Biofilm matrices contain both components in varying proportions of polysaccharides and proteins; combination therapy potentially achieves more complete disruption than single agents (Carter et al., 2021).

**Figure 1:**
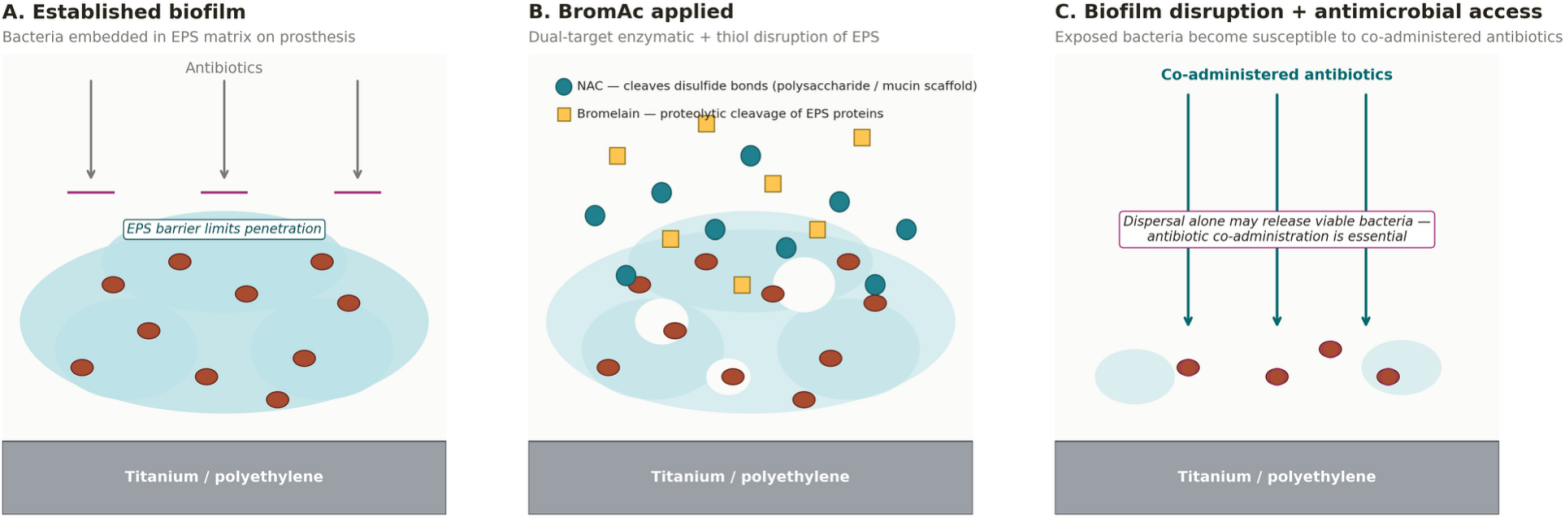
Proposed mechanism of BromAc-mediated biofilm disruption on prosthetic implants. **(A)** Established biofilm: bacteria are embedded within an extracellular polymeric substance (EPS) matrix on the prosthetic surface, conferring resistance to systemic antibiotics through impaired penetration, metabolic heterogeneity, and persister formation. **(B)** BromAc applied: N-acetylcysteine reduces disulfide cross-links within the polysaccharide/mucin component of the matrix, while bromelain proteolytically degrades the protein scaffold; dual-target action disaggregates the biofilm. **(C)** Following disruption, previously embedded organisms are exposed and become susceptible to co-administered antimicrobials; as dispersal alone may release viable planktonic bacteria, antibiotic co-administration is essential.

Additional potential synergies include enhanced penetration (NAC-mediated polysaccharide disruption enhancing bromelain penetration to proteins), broader pathogen spectrum (different organisms emphasise different EPS components), reduced resistance development through dual mechanisms, and complementary anti-inflammatory effects (Carter et al., 2021). The temporal sequence of action remains under investigation, with hypotheses suggesting NAC-mediated polysaccharide disruption may facilitate subsequent bromelain penetration to protein targets. Importantly, no antagonistic interactions have been observed between the two agents, and synergy appears consistent across different bacterial species and biofilm phenotypes (Carter et al., 2021). These mechanistic claims are biologically plausible but should be regarded as hypotheses: the strength of evidence behind each varies considerably, and most evidence rests on single in vitro experiments. The proposed mechanism is summarised in Fig. 1.

The proof-of-concept study examined BromAc against bacterial biofilms on hernia mesh contaminated with three different *Pseudomonas aeruginosa* strains (Carter et al., 2021). Investigators tested NAC alone, bromelain alone, and BromAc combinations at different ratios (Carter et al., 2021). Notably, Carter et al. (2021) observed that NAC alone showed growth enhancement in two of the three *P. aeruginosa* strains, while biofilms were more susceptible to bromelain alone; however, the BromAc combination overcame this limitation, achieving > 80 % debridement across all strains (Carter et al., 2021). Quantitatively, biofilm biomass (measured by crystal violet optical density) was reduced by approximately 82 %–87 % across the three strains relative to untreated controls, with corresponding 1–2 log10 reductions in adherent CFU; the strongest effect was observed at higher bromelain:NAC ratios. Importantly, synergy was observed across three distinct *P. aeruginosa* strains with different biofilm phenotypes, suggesting robust activity. Microscopy confirmed physical disruption of biofilm architecture with exposure of previously embedded bacteria (Carter et al., 2021).

While this study used surgical mesh rather than orthopaedic implants and *P. aeruginosa* rather than staphylococci dominating PJI, the proof of concept is compelling, supporting a dual-mechanism rationale and potential translatability to PJI scenarios (Carter et al., 2021). The observed synergy likely involves sequential matrix disruption, enhanced enzyme penetration through disrupted polysaccharides, reduced enzyme inactivation, and complementary anti-adhesion effects (Carter et al., 2021). It should be noted that the primary proof-of-concept study (Carter et al., 2021) was funded by the manufacturer of BromAc, and independent replication is warranted (Carter et al., 2021). More broadly, the available BromAc evidence base aggregates studies that differ in pathogen (*P. aeruginosa*, *S. aureus*, *S. epidermidis*), substrate (hernia mesh, stainless steel clips, bone screws, titanium/polyethylene discs), exposure time (3 to 24 h), outcome measure (crystal violet OD, CFU recovery, fluorescence microscopy), and BromAc concentration/ratio. These methodological differences materially affect the comparability of reported efficacy figures and are summarised in Table 1; readers should be cautious about pooling or directly comparing results across these studies.

While the Carter et al. (2021) study used surgical mesh and *P. aeruginosa*, more recent preclinical data have demonstrated BromAc activity against *S. aureus* biofilms on surgical metal clips as a surrogate for orthopaedic prostheses. BromAc effectively removed biofilm from all four *S. aureus* strains tested, achieving comparable efficacy to high-dose gentamicin alone, while sequential BromAc–antibiotic treatment reduced bacterial viability across all tested strains (Mucpharm, data on file). In the underlying experiments (provided by the sponsor and not peer reviewed at the time of writing), BromAc alone reduced adherent biofilm biomass by approximately 60 %–75 % across the four strains versus untreated controls, broadly comparable to gentamicin at 1 mg mL^−1^; sequential application of BromAc followed by gentamicin reduced recoverable CFU by approximately 2–3 log10 across all four strains, exceeding either monotherapy. These figures should be interpreted with caution: the data are unpublished and originate from the developer of BromAc; the surrogate substrate (surgical metal clips) does not reproduce the loaded titanium/polyethylene, articulating interface of an arthroplasty; and the assay protocols and replicate numbers are not yet available in peer-reviewed form.

On surgical mesh, BromAc alone reduced *S. aureus* biofilm biomass by 56 %, increasing to 63 % with gentamicin, notably exceeding the biofilm reduction achieved by very high concentrations of antibiotics alone (Mucpharm, data on file).

Against *S. epidermidis*, the most common coagulase-negative staphylococcal pathogen in PJI, all examined BromAc combinations exhibited synergistic effects with near-complete biofilm eradication (Mucpharm, data on file). In the sponsor's dataset, BromAc combined with vancomycin or rifampicin produced ≥ 90 % biofilm biomass reduction and ≥ 3 log10 CFU reductions in *S. epidermidis* on mesh and clip substrates; however, as with the *S. aureus* data above, this evidence is sponsor generated, unpublished, and obtained on non-orthopaedic substrates. It therefore narrows but does not close the gap in peer-reviewed staphylococcal validation on prosthetic materials and should not be relied upon as definitive.

Based on mechanistic rationale and preclinical evidence, BromAc represents biologically plausible but as-yet-unproven adjunctive therapy for PJI, with several potential clinical applications, including as an intraoperative DAIR adjunct, as an ex vivo implant treatment, as a post-operative intra-articular therapy, or in combination with antibiotics for administration. Each of these proposed delivery modes implies a distinct set of unanswered translational questions, contact time, carrier compatibility, intra-articular toxicity, systemic absorption, and formulation stability at the point of use, which would each need to be addressed in dedicated preclinical work before any first-in-human orthopaedic study could be contemplated.

Before clinical translation, the following critical safety questions require investigation: intra-articular toxicity (cartilage effects, synovial inflammation), effects on bone–implant osseointegration, systemic absorption and systemic effects, optimal formulation (NAC : bromelain ratios, pH, osmolality), dosing and exposure timing, and potential bacterial nutrient provision.

**Table 1:** Comparative summary of principal preclinical studies of NAC, bromelain, and BromAc relevant to prosthetic joint infection.

Study	Agent	Pathogen	Substrate	Exposure	Outcome metric	Main finding	Key limitation(s)
Drago 2013	NAC	*S. aureus*, *P. aeruginosa*	Polyethylene, sand-blasted titanium discs	3 h, MIC– 4× MIC	Crystal violet OD, CFU	∼ 50 % biofilm reduction on polyethylene, ∼ 20 % on titanium at MIC	Short exposure, OD does not distinguish between live/dead, limited strains
Jotic 2025	NAC	*Staphylococcus* spp. (chronic rhinosinusitis isolates)	Polystyrene microtitre plate	24 h, 4× – 8× MIC	CFU eradication	Eradication in 42.6 % ( 4× MIC) and 81.5 % ( 8× MIC) of isolates	Non-orthopaedic substrate and pathogen niche, no implant material
Manoharan 2020	NAC + antibiotic	MRSA, MSSA	Polystyrene, in vitro	24 h, 30 mM NAC	Biomass + CFU	≥90 % biofilm disruption with combined treatment	In vitro only, not on prosthetic material
Watters 2016	Bromelain	*S. aureus*	Plasma-supplemented wound biofilm model	Variable; up to 24 h	Biomass	Up to 98 % biomass reduction	Wound-care model, not implant-loaded biofilm
Bratton 2025	Bromelain ± scrubbing	MRSA (single strain)	Cortical bone screws	Short in vitro contact	Crystal violet OD	Significant OD reduction, greatest with powder + scrubbing	OD only (live/dead not separated), single strain, non-articulating substrate
Carter 2021	NAC, bromelain, BromAc	*P. aeruginosa* (3 strains)	Polypropylene hernia mesh	24 h	Crystal violet OD; CFU	BromAc ∼ 82 %–87 % biomass reduction, 1–2 log10 CFU drop, NAC alone enhanced growth in 2/3 strains	Mesh ≠ articulating prosthesis, pathogen not dominant in PJI, manufacturer funded
Mucpharm data on file	BromAc ± gentamicin	*S. aureus* (4 strains)	Stainless steel surgical clips	Not specified	Biomass, CFU	∼ 60 %–75 % biomass reduction, gentamicin comparable, + 2–3 log10 CFU with sequential antibiotic	Unpublished sponsor data, non-orthopaedic loading, competing interest
Mucpharm data on file	BromAc ± vancomycin/ rifampicin	*S. epidermidis*	Surgical mesh, metal clips	Not specified	Biomass, CFU	≥90 % biomass reduction, ≥ 3 log10 CFU reduction with antibiotic	Unpublished sponsor data, not yet peer reviewed, non-implant substrate
Mucpharm pilot in vivo (data on file)	BromAc (subcutaneous)	*S. aureus*	Subcutaneous mesh/Liga-clip mouse model	5 d SC dosing	Animal safety, biofilm load	No adverse events, bromelain 3 mg kg^−1^ + NAC 300 mg kg^−1^ tolerated	Subcutaneous (not intra-articular), no loaded prosthesis, sponsor data

Abbreviations: CFU, colony-forming unit; MIC, minimum inhibitory concentration; and OD, optical density.

## 9 Discussion

PJI remains a devastating complication with suboptimal treatment outcomes, particularly for implant-retention strategies. The central role of biofilm formation conferring antimicrobial resistance necessitates novel approaches disrupting biofilm architecture.

NAC and bromelain individually demonstrate antibiofilm activity through complementary mechanisms, as discussed earlier. The combination of bromelain and NAC achieve superior biofilm debridement ( > 80 %) compared to either agent alone (Carter et al., 2021), as quantified by crystal violet biomass reduction in the Carter et al. (2021) proof-of-concept study; however, as detailed in Sect. 7 and Table 1, this figure is derived from *P. aeruginosa* biofilms on hernia mesh and should not be extrapolated, without qualification, to staphylococcal biofilms on titanium or polyethylene.

Preclinical evidence on orthopaedic prosthetic materials specifically demonstrates NAC's capacity to disaggregate established staphylococcal biofilms on titanium and polyethylene (Drago et al., 2013) and bromelain's ability to achieve near-complete biofilm removal on orthopaedic implants (Bratton et al., 2025). Even this narrower evidence is, however, limited to short exposure times, biomass-only readouts, single strains, and non-loaded substrates, and the published BromAc data on prosthetic-grade orthopaedic materials remain absent, and the existing staphylococcal data are sponsor generated and not yet peer reviewed.

The BromAc approach offers several theoretical strengths as a potential adjunctive therapy for prosthetic joint infection. It has a strong mechanistic rationale, simultaneously targeting distinct biofilm components, and preclinical proof of concept has been demonstrated in multiple experimental models, albeit in predominantly non-orthopaedic substrates. Long-standing clinical use of bromelain and N-acetylcysteine in other indications provides established safety profiles, and the combination is likely to be cost-effective compared with complex revision arthroplasty. In addition, BromAc has broad applicability across different pathogens and implant materials and can, in principle, be delivered via multiple routes, including intraoperative application, irrigant solutions, or incorporation into local carriers such as cement. Importantly, BromAc has already been administered to over 100 patients via intratumoral and intraperitoneal injection for the treatment of pseudomyxoma peritonei, with a manageable safety profile (Mucpharm, data on file). Phase I first-in-human and Phase III clinical trials have been conducted, establishing a human safety precedent that may facilitate translation to orthopaedic applications. This precedent applies to intraperitoneal and intratumoural delivery; it does not, by itself, establish intra-articular safety, where the cartilage, synovium, and bone–implant interface are uniquely vulnerable.

Important limitations and knowledge gaps remain before BromAc can be adopted clinically for PJI. Current evidence is largely derived from non-PJI settings, with very limited data specifically in prosthetic joint infection. While no in vivo orthopaedic animal model using actual prosthetic implants under physiologic loading conditions has been completed, a pilot animal study has established a subcutaneous implant-associated infection model using polypropylene mesh and stainless steel Liga clips with *S. aureus* biofilm and confirmed the safety of subcutaneous BromAc administration (bromelain 3 mg kg^−1^, acetylcysteine 300 mg kg^−1^) over 5 d with no adverse events (Mucpharm, data on file). Across 21 preclinical animal experiments, BromAc has demonstrated a consistently favourable safety profile via intraperitoneal, subcutaneous, intravenous, and inhaled routes of administration. None of these routes is equivalent to intra-articular delivery in an implanted, weight-bearing joint, and the safety database for intra-articular BromAc is, at present, empty. The translation from hernia mesh (Carter et al., 2021) and stainless steel hardware (Bratton et al., 2025) to the unique environment of the synovial joint with titanium/polyethylene prostheses requires dedicated investigation. Intra-articular safety with respect to cartilage, synovium, and osseointegration is unknown, and optimal formulation, concentration, and dosing regimens have not been defined. Formulation stability is a particular concern: NAC undergoes thiol autoxidation in aqueous solution with measurable loss of activity within hours under ambient conditions (Sect. 5.1), and bromelain is subject to autodigestion; both will likely require point-of-use reconstitution, preservative-free buffering, and oxygen-protective packaging for any intra-articular application. Initial proof-of-concept studies focused primarily on *Pseudomonas aeruginosa*; however, more recent preclinical data have demonstrated BromAc efficacy against *S. aureus* (four strains, including on metal clip surrogates for prosthetic materials) and *S. epidermidis*, with synergistic biofilm eradication observed across all staphylococcal species tested (Mucpharm, data on file). These staphylococcal datasets are not yet peer reviewed and are sponsor generated; peer-reviewed, independent staphylococcal validation on titanium and polyethylene substrates therefore remains the single most important unmet evidence requirement. Nevertheless, further investigation against clinical PJI isolates, including MRSA and polymicrobial biofilms, remains necessary.

Translation to clinical trials requires systematic completion of several preclinical milestones. Priority investigations include dose–response characterisation against dominant PJI pathogens (including MRSA and coagulase-negative staphylococci), synovial fluid pharmacokinetic profiling to establish residence time and effective concentrations, cartilage and synovial tissue tolerance studies in ex vivo human models, and large-animal proof-of-concept studies using actual orthopaedic prosthetic materials under physiologic loading conditions. Specifically, a structured in vivo programme should comprise, in our view, the following sequential studies before any first-in-human orthopaedic trial. First, a small-animal (rodent) implanted titanium or polyethylene insert model of established * S. aureus* and *S. epidermidis* biofilm, with co-primary endpoints of (a) viable CFU recovery from sonicated explanted implants and (b) histological assessment of synovial inflammation and cartilage architecture, comparing intra-articular BromAc + antibiotic against antibiotic alone and against saline irrigation. Second, a large-animal (rabbit or sheep) loaded prosthesis model with the same endpoints plus dynamic synovial fluid pharmacokinetic sampling, contact time characterisation, and longer-term assessment of osseointegration on retrieved implants. Third, dedicated ex vivo human cartilage and synovium tolerance studies across the BromAc concentration range, including chondrocyte viability, matrix metalloproteinase release, and proteoglycan content. Fourth, formulation-stability studies under operating-theatre-realistic conditions (ambient O_2_ exposure, reconstitution-to-use intervals of 0–6 h, with quantitation of free thiol and proteolytic activity over time). Only on the basis of consistent positive efficacy and acceptable safety across all four streams, and ideally with independent replication outside the sponsor's laboratory, would a Phase I intra-articular study in elective DAIR be defensible.

## 10 Conclusions

Prosthetic joint infection remains a serious complication of total joint arthroplasty with suboptimal outcomes, particularly for implant-retention strategies. Biofilm formation confers marked antimicrobial resistance, explaining conventional treatment failures.

N-acetylcysteine and bromelain individually demonstrate substantial antibiofilm activity through complementary mechanisms. The combination (BromAc) has been shown to achieve > 80 % biofilm debridement in the principal *P. Aeruginosa* hernia mesh proof-of-concept study, exceeding either agent alone. BromAc represents a mechanistically rational, cost-effective adjunctive therapy for PJI, warranting systematic, independent investigation. However, the current evidence base is predominantly preclinical, frequently obtained on non-orthopaedic substrates and pathogens, and partly drawn from unpublished sponsor data; several critical translational questions – peer-reviewed staphylococcal validation on prosthetic materials, formulation stability of NAC and bromelain in aqueous solution, intra-articular cartilage and osseointegration safety, and demonstration of efficacy in a loaded in vivo orthopaedic implant model – remain unresolved. Clinical adoption should not be considered until these gaps are closed.

If the necessary preclinical programme outlined above ultimately supports clinical evaluation, BromAc could, in principle, improve DAIR success rates and preserve implant retention advantages, with knock-on benefits for PJI-associated morbidity and healthcare burden and possible applicability to other biofilm-associated implant infections. On present evidence, however, BromAc should be regarded as a promising but unvalidated adjunct, and any clinical use outside a properly designed and regulated trial is not currently justified.

## Data Availability

No original data were generated in this study. All source material is cited within the reference list and is publicly accessible via the respective publishers or PubMed/MEDLINE.
